# Synthesis of Some New Quinazolinone Derivatives and Evaluation of Their Antimicrobial Activities

**Published:** 2012

**Authors:** Ghadamali Khodarahmi, Elham Jafari, Gholamhossein Hakimelahi, Daryoush Abedi, Marzieh Rahmani Khajouei, Farshid Hassanzadeh

**Affiliations:** a *Department of Medicinal Chemistry, Faculty of Pharmacy and Pharmaceutical Sciences, Isfahan University of Medical Sciences, Isfahan, Iran.*; b *Isfahan Pharmaceutical Sciences Research Center, Isfahan, Iran. *; c *Institute of Chemistry, Academia Sinica, Nankang, Taipei, Taiwan.*; d *Department of Microbiology, Faculty of Pharmacy and Pharmaceutical Sciences, Isfahan University of Medical Sciences, Isfahan, Iran.*

**Keywords:** Fused quinazolinons, Deoxyvasicinone, Benzoxazinone, Antibacterial activity, Antifungal activity

## Abstract

Wide range of quinazolinone biological properties including: antibacterial, anticancer, and anti-inflammatory activities encouraged us to synthesis some fused quinazolinone derivatives.

Anthranilic acid was condensed with chloro acylchloride followed by dehydration to form the benzoxazinone intermediate; subsequent addition of an amine provided the fused quinazolinones. Deoxyvasicinone which was previously synthesized by a multi step complex reactions was prepared in three steps using the following procedure:

Log *P *values of the compounds were measured using the shake flask method in octanol/water solvent system.

The synthesized compounds were evaluated against six strains of bacteria (three Gram-positive and three Gram-negative) and three strains of fungi.

Overall results of antimicrobial tests showed that the compounds had better bacteriostatic activity against Gram-negative bacteria. The obtained results of MBC revealed that these compounds had more significant bacteriostatic than bactericidal activities. Almost all of the screened compounds showed good activity against *C. albicans *and *A. niger. *The obtained results of MFC indicated that these compounds had more significant fungistatic than fungicidal activities.

## Introduction

Quinazolinones (benzopyrimidine derivatives), are compounds with wide spectrum of biological activities, including: anti cancer, anti convulsant, anti inflammatory, anti tubercular and anti bacterial effects (1-7).

Several biological activities were reported for fused tricyclic quinazoline derivatives (8, 9) among which deoxyvasicinone is highly interesting and has been investigated for its antimicrobial and anti cancer effects (10, 11). In addition, deoxyvasicinone is a very important key intermediate for the synthesis of various natural products such as vasicinone and isaindigotone (11). The present study plans to synthesize some new tricyclic quinazolinone derivatives to be evaluated for antimicrobial activities. Deoxyvasicinone which was previously synthesized by multi step complex reactions or application of microwave irradiation (10-15) is also prepared here in a simple procedure.

A highly employed method for 4(3*H*)-quinazolinone synthesis is based on acylation of anthranilic acid with acyl chloride. Subsequent ring closure with acetic anhydride afford corresponding 1,3-benzoxazin-4-one (benzoxazinone) which will be treated with different amines to give 4(3*H*)-quinazolinone derivatives (16). Here a number of fused quinazolinone derivatives including deoxyvasicinone were synthesized using this procedure. In this method chloro acyl chloride was used instead of acyl chloride. The second ring closure was achieved simultaneously to produce fused quinazolinone derivatives in acceptable yields. Synthesized compounds were evaluated against six strains of bacteria (three Gram-positive and three Gram-negative) and three strains of fungi.

## Experimental

*Chemistry*

All solvents and the chemicals used in this study were purchased from Merck Co. (Merck, Germany). Melting points were determined in open capillaries using electrothermal 9200 melting point apparatus and are uncorrected. ^1^H-NMR spectra were recorded on a Bruker 400 MHz spectrometer and the chemical shifts are expressed as *δ *(ppm) with tetramethylsilane as internal standard. Mass spectra were recorded on Shimadzu Mass spectrometer. IR spectra were recorded with a WQF -510 FT-IR spectrophotometer.

*Synthesis of 2-(4-chloro-butanamido)-benzoic acid (2)*

4-Chlorobutyrylchloride (0.55 mole) was added drop-wise to a stirring solution of anthranilic acid (0.5 mole) in dimethyl formamide (250 mL). The mixture was poured into water to produce a precipitate which was collected by filtration, as a white solid (65%), m.p. 112.5–113οC (Found: M 241, C_11_H_12_Cl NO_3_ requires 241), ν_max_, 3126 (NH), 2873 (C- H), 1689, 1647 (C=O), 779 (C-Cl) cm^-1^; δ_H_ (400MHz ; CDCl_3_) 13.55 (1H, bs, COOH), 11.1(1H, s, NH), 8.41 (1H, d, *J *= 8 Hz, H-3 Ar), 7.92 (1H, d, *J *= 8 Hz, H-6 Ar), 7.51 (1H, t, *J *= 8 Hz, H-4 Ar), 7.1 (1H, t, *J *= 8 Hz, H-5 Ar), 3.71 (2H, t, *J *= 8 Hz, NH-CO-CH_2_-CH_2_-CH_2_-Cl), 2.51 (2H, t, *J *= 8 Hz, NH-CO-CH_2_-CH_2_-CH_2_-Cl ), 2.1 (2H, qui, *J *= 8 Hz, NH-CO-CH_2_-CH_2_-CH_2_-Cl).

*Synthesis of 2-(3-chloro-propyl)-4H-benzo[d] [1, 3] oxazin-4-one (3)*

Compound 2 (0.25 mole) was dissolved in acetic anhydride (180 mL) and heated for one hour with vigorous stirring. The excess of acetic anhydride was removed by distillation under reduced pressure. The residue was cooled and the product crystallized. The product was triturated with *n*-hexane and isolated by filtration to yield compound 3 as yellow crystals (62%), m.p. 100.8 – 101.5 οC, ν_max_, 3059 (C-H) 1770 (C=O) 1640 (C=N) cm^-1^ ; δ_H_ (400MHz ; CDCl_3_) 8.25 (1H, d, *J *= 6.9 Hz, H-5 Ar), 7.85 (1H, d, *J *= 6.9 Hz, H-8 Ar ), 7.6 (2H, m, H-6 and H-7 Ar), 3.75 (2H, t, *J *= 6.2 Hz, COO-C-CH_2_-CH_2_-CH_2_-Cl), 2.9 (2H, t, *J *= 6.2 Hz, COO-C-CH_2_-CH_2_-CH_2_-Cl), 2.35 (2H, qui, *J *= 6.2 Hz, COO-C-CH_2_-CH_2_-CH_2_-Cl).

*Synthesis of 1,2,3,4-tetrahydropyridazino [6,1b] quinazolin-10-one (4).*

Compound 3 (0.15 mole) and excess of hydrazine hydrate were refluxed in ethanol for 2 h. The reaction was monitored by TLC and after complete consumption of compound 3 the mixture was purified by column chromatography on silica gel using an eluent of CHCl_3_-MeOH 49:1. A fraction containing compound 4 was collected as white crystals (40%), m.p. 152.7 –153.7οC, (MS: m/z (%): 201(M^+^, 100), 173 (37.5), 146 (8.3), 119 (16.6) , C_11_H_11_N_3_O M.W. 201) ν_max_, 3263 (N-H) 2974-2883 (C-H), 1662 (C=O), 1608 (C=C, Ar) cm^-1^; δ_H_ (400MHz ; CDCl_3_) 8.27(1H, d, *J *= 8.5Hz, H-9 Ar), 7.74 (1H, t, *J *= 8.4Hz, H-7 Ar ), 7.68 (1H, d, *J *= 8.4 Hz, H-6 Ar ) ,7.47 (1H, t, *J *= 8.5Hz, H-8 Ar ), 7.14 (1H, s, N-H), 3.29 (2H, t, *J *= 7.2 Hz, N=C-CH_2_-CH_2_- CH_2_), 3.07 (2H, t, *J *= 7.2 Hz, N=C-CH_2_-CH_2_-CH_2_), 2.23 (2H, qui, *J *= 7.2 Hz, N=C-CH_2_-CH_2_-CH_2_).

*Synthesis of 2,3-dihydropyrrolo [2,1-b] quinazoline-9(1H)-one ( deoxyvasicinone) (5)*

Compounds 3 (0.15 mole) and excess of ammonium acetate was refluxed in ethanol for 2 h. After complete consumption of compound 3 the mixture was purified by column chromatography on silica gel using an eluent of CHCl_3_-MeOH 19:1 to give compound 5 as white crystals (30%), m.p. 198.7- 199.5οC, (lit 196 - 198 οC) (8), (MS: m/z (%):186, (M^+^, 100) 158 (79), 132 (41.6), C_11_H_10_N_2_O M.W. 186) ν_max_, 3059 (C-H Ar), 2927 (C-H), 1678 (C=O), 1635 (C=N), 1603 (C=C, Ar), 1259 (C-N) cm^-1^; δ_H_ (400MHz ; CDCl_3_) 8.34(1H, dd, *J *= 8.4 Hz, *J *= 1.2 Hz, H-8 Ar), 7.72 (1H, dt, *J *= 8.4Hz, *J *= 1.2Hz, H-6 Ar ), 7.47 (1H, t, *J *= 8.5Hz, H-7 Ar ) , 7.21 (1H, d, *J *= 8.5Hz, H-5 Ar ), 4.25 (2H, t, *J *= 7.6 Hz, N=C-CH_2_-CH_2_-CH_2_), 3.2 (2H, t, *J *= 7.6Hz, N=C-CH_2_-CH_2_-CH_2_), 2.42 (2H, qui, *J *= 7.6Hz, N=C-CH_2_-CH_2_-CH_2_).

*Synthesis of 3, 3-dibromo-2,3-dihydropyrrolo [2,1-b] quinazolin-9(1H)-one (6).*

To a three-neck round-bottom flask equipped with a dropping funnel, was added compound **5 **(0.1 mole), anhydrous sodium acetate (10 g) and glacial acetic acid (130 mL). Bromine (16 g) in acetic acid (10 mL) was added drop wise to the solution in 1-2 h. After complete addition of bromine, the mixture was poured into ice water; the precipitated product was isolated by filtration to obtain compound 6 As light brown crystals (29%), mp 145οC (decomposed). MS: m/z (%): 344 (M^+^,16), 346 (32), 264 (41) C_11_H_8_Br_2_N_2_O M.W. 344, ν_max_, 2991-2951(C-H), 1665(C=O), 1601 (C=C, Ar) cm^-1^; δ_H_ (400MHz ; CDCl_3_) 8.41(1H, d, *J *= 8Hz, H-8 Ar), 7.79 (1H, t, *J *= 8Hz, H-6 Ar), 7.58 (1H, t, *J *= 8Hz, H-7 Ar), 7.31 (1H, d, *J *= 8Hz, H-5 Ar), 4.25 (2H, t, *J *= 7.6Hz, CBr_2_-CH_2_-CH_2_), 3.47 (2H, t, *J *= 7.6Hz, CBr_2_-CH_2_-CH2).

*Synthesis of 2-(3-chloropropanamido) benzoic acid (7)*

3-Chloropropionyl chloride (0.55 mole) and anthranilic acid (0.5 mole) were reacted according to the procedure explained for 2 to give 7 as a white solid (65%), m.p. 148.3-148.6οC (Found: M 227.5 C_10_H_10_ClNO_3 _requires 227.5) ν_max_, 3303 (NH), 2972-2873 (C-H), 1678, 1700 (C=O), 1600, 1475(C=C Ar), 756 (C-Cl) cm^-1^; δ_H_ (400MHz ; CDCl_3_) 13.55 (1H, bs, COOH), 11.45 (1H, s, NH), 8.57 (1H, d, *J *= 6.9 Hz, H-3 Ar), 8.1 (1H, d, *J *= 6.9 Hz, H-6 Ar), 7.45 (1H, t, *J *= 6.9 Hz, H-4 Ar), 7.1 (1H, t, *J *= 6.9 Hz, H-5 Ar ), 3.85 (2H, t *J *= 6.6 Hz, NH-CO-CH_2_ -CH_2_-Cl), 2.9 (2H, t, *J *= 6.6 Hz, NH-CO-CH_2_ -CH_2_-Cl).

*Synthesis of 2-(2-chloroethyl)-4H-benzo [d] [1,3] oxazin-4-one (8)*

Compound **7 **(0.25 mole) was dissolved in acetic anhydride (180 mL) and heated for one hour with vigorous stirring. The solvent was removed through distillation under reduced pressure. The residue was used directly for the next step without further purification.

*Synthesis of 3-amino-2-(3-ethoxy-ethyl) quinazoline-4(3H)-one (9) and 2, 3-dihydropyrazolo [5, 1-b] quinazolin-9(1H)-one (10).*

The benzoxazinone 8 (0.15 mole) and excess of hydrazine-hydrate were dissolved in ethanol and refluxed for 2 h. The mixture was purified by column chromatography on silica gel using an eluent of CHCl_3_-MeOH 49:1 to give compounds 9 and 10.

(9): white crystals, yield (33% ), m.p. 113.5-114.1 οC (MS: m/z (%): 233 (M^+^, 8), 204 (100), 160 (16), 189 (50), 119 (20), C_12_H_15_N_3_O_2_ M.W. 233) ν_max_,3336- 3278 (NH_2_) 2976-2870 (C-H), 1676 (C=O), 1601 (C=C, Ar), 1120 (C-O-C) cm^-1^; δ_H_ (400MHz ; CDCl_3_) 8.27(1H, d, *J *= 8.5Hz, , H-5 Ar ), 7.75 (1H, t, *J *= 8.4Hz, H-7 Ar), 7.68 (1H, d, *J *= 8.5Hz H-8 Ar ), 7.47 (1H, t, *J *= 8.5Hz, H-6 Ar ), 5.57 (2H, s, NH_2_), 3.94 (2H, t, *J *= 4.8Hz, N=C-CH_2_-CH_2_-O), 3.53 (2H, qua, *J *= 6.8 Hz, O-CH_2_- CH_3_), 3.37 (2H, t, *J *= 4.8Hz, N=C-CH_2_-CH_2_-O), 1.18 (3H, t, *J *= 6.8, CH_3_).

(10): white crystals, yield **(**37 %), m.p. 160.8-161.5 οC (MS: m/z (%): 187 (M^+^, 100), 160 (14), 145 (4), C_10_H_9_N_3_O M.W. 187) ν_max_, 3234 (NH), 2997-2931 (C-H), 1650 (C=O), 1626 (C=N), 1605 (C=C, Ar) cm^-1^; δ_H_ (400MHz; CDCl_3_), 8.28 **(**1H, d, *J *= 8 Hz, H-8 Ar), 7.73 (1H, t, *J *= 8 Hz, H-6 Ar), 7.68 (1H, d, *J *= 8 Hz, H-5 Ar) 7.47 (1H, t, *J *= 8 Hz, H-7 Ar ), 5.8 (1H, s, NH) 3.71(2H, t, *J *= 8 Hz, N=C-CH_2_-CH_2_), 3.41 (2H, t, *J *= 8 Hz, N=C-CH_2_-CH_2_).

*Synthesis of 2-(3-chloro-propyl)-2-hydroxy-3-phenyl-2,3-dihydroquinazolin-4(1H)-one (11)*

Compound 3 (0.15 mole) was reacted with aniline (0.16 mole) in toluene and refluxed for 5 h. After complete consumption of benzoxazinone, solvent was distilled off under reduced pressure. The mixture was purified by column chromatography on silica gel using eluent of CHCl_3_-MeOH 40:1.Compound 11 was isolated in 28% yield as white crystals, m.p. 149.8-150.1 οC, (MS: m/z (%): 316 (M^+^, 4), 250 (8), 235 (69), 224 (6), 146 (14), C_17_H_17_ClN_2_O_2_ M.W. 316), ν_max_, 3240 (NH), 3143-3095 (C-H Ar), 2925 (C-H), 1653 (C=O), 1608 (C=C Ar), cm^-1^; δ_H_ (400MHz;CDCl_3_) 10.84 (1H, S, NH), 8.62 (1H,d, *J *= 8.4 Hz, CO-C=CH-CH=CH-CH=CH-NH), 7.94 (1H,S,OH), 7.64 (1H, d *J *= 8 Hz, CO-C=CH-CH=CH-CH=C-NH), 7.6 (2H, d, *J *= 8 Hz, CO-N-C-CH=CH-CH=CH-CH), 7.54 *(*1H, t, *J *= 8 Hz, CO- C=CH-CH=CH-CH=C-NH), 7.43 (2H, t, *J *= 8 Hz, CO-N-C-CH=CH-CH=CH-CH), 7.25(1H, t, *J *= 8 Hz, CO-N-C-CH=CH-CH=CH-CH ), 7.17(1H, t, *J *= 8 Hz, CO-C=CH-CH=CH-CH=C-N), 3.66 (2H, t, *J *= 6.4 Hz, NH-C (OH)-CH_2_-CH_2_-CH_2_-Cl), 2.62 (2H, t, *J *= 7.2 Hz, NH-C (OH)- CH_2_-CH_2_-CH_2_-Cl), 2.22 (2H, qui, *J *= 6.4 Hz, NH-C (OH)-CH_2_-CH_2_-CH_2_-Cl).

*Synthesis of 2-(4-oxo-4H-benzo [d] [1,3] oxazin-2-yl)-benzoic acid (12)*

Anthranilic acid 1 (0.04 mole) was treated with phetalic anhydride (0.04 mole) in glacial acetic acid for 5 h. After completion of the reaction, solvent was distilled off under reduced pressure, the residue was recrystallized from acetone to obtain compound 12 as white crystals (48%) m.p. 220.4-221.8 οC (MS: m/z (%): 267 (M^+^, 5), 224 (2.6), 223 (13), 221 (10), C_15_H_9_NO_4_ M.W. 267) ν_max_, 3178 (OH COOH), 3091 (C-H Ar), 1720, 1701 (C=O), 1603 (C=C, Ar) cm^-1^ ; δ_H_ (400MHz; CD_3_OD), 8.17 (1H, d, *J *= 7.6 Hz, CO-C=CH-CH=CH-CH=C-N), 7.97 (2H, m, COO-C-C-CH=CH-CH=CH-C-COOH), 7.88 (2H, m, COO-C-C-CH=CH-CH=CH-C-COOH), 7.75 (1H, t, *J *= 7.6 Hz, CO-C=CH-CH=CH-CH=C-N), 7.62 (1H, t, *J *= 7.6 Hz, CO-C=CH-CH=CH-CH=C-N), 7.46 (1H, d, *J *= 8 Hz, CO-C=CH-CH=CH- CH=C-N).

*Synthesis of 2-Benzyl*-isoindoline-1, 3-Dione (13)

Compound 12 (0.005 mole) and benzyl amine (0.005 mole) refluxed in toluene for 5 h.The precipitated product was collected by filtration. Ethylene glycol and NaOH (0.01 g) were added to residue and heated in an oil bath for 2h.The crystals were filtered off to provide compound 13 as brown crystals (42% ) m.p. 109.5-110.7 ^ο^C (MS: m/z (%): 237 (M^+^, 100), 219 (33), 160 (8), C_15_H_11_NO_2_ M.W. 237), ν_max_, 3060 (C-H Ar), 2947 (C-H), 1765-1712 (C=O) cm^-1^ ; δ_H_ (400MHz; CDCl_3_) 7.85 (2H, dd, *J *= 2.8 Hz, *J *= 5.2 Hz, CO-C=CH-CH=CH-CH=C-CO), 7.71 (2H, dt, *J *= 2.8 Hz, *J *= 5.2 Hz CO-C=CH-CH=CH-CH=C-CO), 7.44 (2H, d, *J *= 7.2 Hz, CO-N-CH_2_-C=CH-CH=CH-CH=CH), 7.33 (2H, t, *J *= 7.6, CO-N-CH_2_-C=CH-CH=CH-CH=CH), 7.28 (1H, t, *J *= 7.6, CO-N-CH_2_-C=CH-CH=CH-CH=CH), 4.86 (2H, s, CH_2_).

*Log P measurement*

Log *p *measurement is a useful parameter to understand the lipophilicity of drug molecules. The shake flask method is the usual way for measuring log *p *values. The UV absorbance of an aqueous solution of a compound is measured before and after being shaken together with a known volume of octanol. One advantage of the method is that the appearance of compound in the octanol may be checked against the disappearance from the aqueous phase. It is very important to pre-saturate the solvents in prolonged shake-flask experiments (17, 18).

*Determination of partition coefficients using the shake flask method*

Partition coefficients (K_part_) of the compounds were determined using the shake flask method. The two phases used in determination were tris buffer (50 mM, pH 7.4, prepared using distilled water) and 1-octanol, each of which was pre-equilibrated with the other phase before use (the solubility of water in 1-octanol is 2.3 M) (17, 18) . The synthesized compounds were dissolved in tris buffer to obtain (10^-4^ M) stock solutions. The relationship between absorbance and concentration of samples (10^-5^, 2×10^-5^, 4 ×10^-5^, 6×10^-5^, 8×10^-5^) were found linear according to beer lambert law. A known volume (normally 10-50 ml) sample of the solution was stirred vigorously with a suitable volume of 1-octanol in a glass vessel for 1 h. The two layers were separated by centrifugation for 5 min. An aliquot of the aqueous layers was then carefully removed using a glass Pasteur pipette ensuring that the sample was not contaminated with 1-octanol. The absorbance of the sample was measured as above and the partition coefficient was then calculated using the following formula:

$K_{\mathrm{part}}=\frac{A_{1}-A_{2}}{A_{2}}\times \frac{V_{w}}{V_{o}}$                     (Equation 1)

A_1_ = Absorbance reading in the aqueous layer before partitioning. A_2_ = Absorbance reading in the aqueous layer after partitioning. V_W_ = Volume of aqueous layer used in partitioning.

V_O_ = Volume of 1-octanol layer used in partitioning. For each compound, the experiment was repeated at least three times for the calculation of a mean K _part_ value and standard deviation. The results are shown in Table 1.

**Table 1:** Log *p *results

**λ**_max_ = **225.9 No.**	**Log ** *p * **(mean ± SD)**
**4**	0.97 **± **0.0 2
**5**	0.85 **± **0.03
**6**	1.02 **± **0.03
**9**	0.7 **± **0.01
**10**	0.68 **± **0.04
**11**	2.1 **± **0.09
**12**	0.45 **± **0.05
**13**	2.1 **± **0.1

*Antimicrobial activity*

Minimum inhibitory concentration (MIC) was determined by micro plate alamar blue assay (MABA) method. Tested bacteria were three Gram-positive bacteria: *(Staphylococcus aureus *PTCC 1337, *Bacillus subtilis *PTCC 1023*, Listeria monocitogenes *PTCC 1165*) *and three Gram-negative bacteria*: (Escherichia coli *PTCC 1338, *Pseudomonas aeruginosa *PTCC 1074, *Salmonella entritidis *PTCC 1091) obtained from Persian Type Culture Collection (PTCC). Tested fungi were one yeast-like fungus *(Candida albicans *PTCC 5027) and two molds *(Aspergillus niger *PTCC 5021 and *Aspergillus flavous *PTCC 5003) obtained from PTCC. Sabouraud dextrose agar was used to culture fungal strains and Mueller Hinton agar was used to culture bacterial strains. The inocula of microorganisms (1.5 × 10^8^ CFU/mL) were prepared from cultures and suspensions were adjusted to 0.5 Mc Farland standard turbidity. Synthesized compounds were dissolved in DMSO (0.5 mL) and diluted with water up to 1 mL to obtain concentration of 5120 μg/mL as stock solutions. The serial dilution method was used to obtain 2560 to 320 μg/mL concentrations (19, 20).

Mueller-Hinton broth was used as medium for bacterial growth. 20 μL of each concentration were distributed in 96-well plates with the exception of those wells acting as growth control (contain microorganisms and culture media) and positive control (contain microorganisms and standard antibiotic). After adding Alamar Blue^®^ reagent (20 μL) to all of the 96 wells total volume in each well became 200 μL. The final concentrations of compounds were (512-32 μg/mL) and the final concentrations of inocula were 1.5 × 10^4 ^for bacteria and 1.5 × 10^5^ for fungi. Plates were covered and sealed with parafilm and incubated for 24 h at 37°C. The MIC was defined as the lowest concentration, which prevented a color change from blue to pink (20, 21). Ciprofloxacin was used as standard antibacterial drug. The same method except for some modifications was used for the antifungal studies. The incubation time was 48 h at 25°C for fungi. Ketoconazole was used as standard antifungal agent. RPMI 1640 medium was used as medium for fungi (20).

Following a broth microdilution MIC test, from each well that shows no growth, contents were removed and spreaded onto mueller Hinton agar plates for bacteria and sabouraud dextrose agar for fungi to determine MBC and MFC results. The plates were incubated for 24 h at 37°C for bacteria and 25°C for fungi (20).

## Results and Discussion

**Figure 1:**
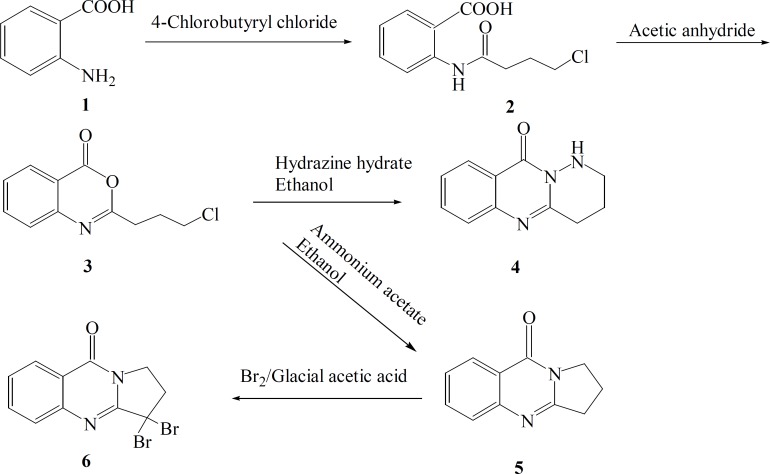
General reaction scheme for the synthesis of the target compounds 4, 5 and 6

**Figure 2:**
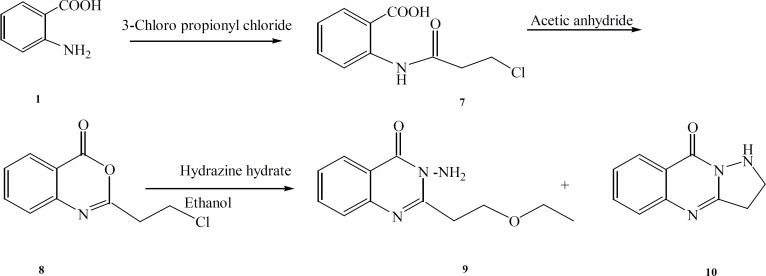
General reaction scheme for the synthesis of the target compounds 9 and 10

**Figure 3:**
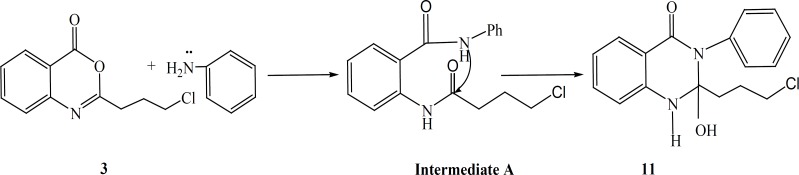
General reaction scheme for the synthesis of the target compounds 11

The synthetic pathways to the intermediates and final compounds (2-10) are presented in Figures 1 and 2. Briefly anthranilic acid was condensed with chloro acylchloride to produce N-acyl antranilic acid. First ring closure and subsequent dehydration were performed with acetic anhydride to form the benzoxazinone intermediate. Finally addition of hydrazine hydrate or ammonium acetate provided the fused quinazolinones (4, 5 and 10). Addition of aniline instead of simple amines resulted in production of compound 11 as presented in Figure 3.

In production of compound 4, hydrazine-hydrate acts as a nucleophile and attacks the carbonyl group of cyclic ester due to its alpha effect. Simultaneous nucleophilic attacks of carbohydrazine nitrogens to the carbonyl group of amide and methylen chloride end group of the side chain resulted in the production of the tricyclic product 4.

In preparation of deoxyvasicinone 5, ammonia acts as a nucleophile and attacks to the carbonyl group of the cyclic ester. Nucleophilic attacks of amine to carbonyl group of amide and methylene chloride end group of the side chain afforded the second tricyclic compound 5 (Figure 1).

The benzoxazinone 8 and excess of hydrazine-hydrate were reacted in ethanol and the reaction mixture was fractionated by column chromatography. Compound 9 is a result of nucleophilic attack of the solvent (ethanol) instead of NH_2 _to the methylene chloride end group of the side chain (Figure 2). Compound 10 which is a five membered ring analogue of 4 was produced by similar mechanism explained in the production of compound 4.

.

**Figure 4:**
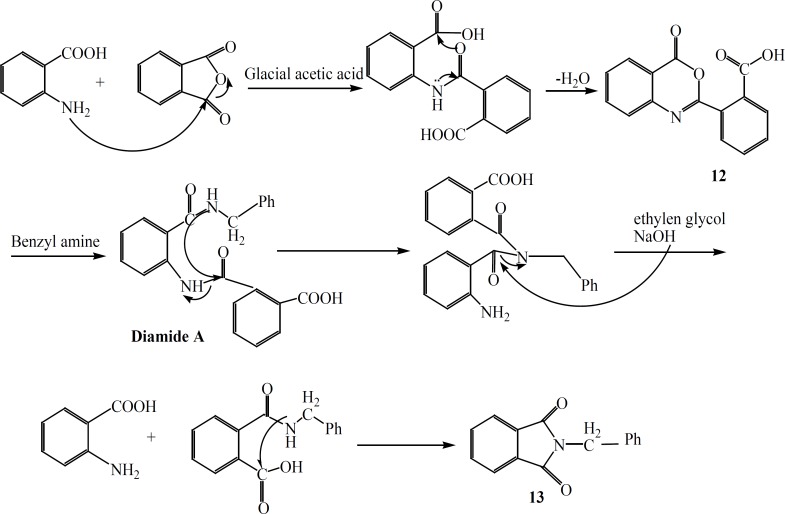
The reaction mechanism to produce compound 13

In an attempt for the production of a quinazolinone derivatives substituted at position 2 and 3 with benzoic acid and benzyl amine, respectively, surprisingly compound 13 (Figure 4) was obtained. The probable mechanism for production of this unexpected compound 13 is shown in Figure 4.

In conclusion application of chloroacyl chlorides instead of acyl chlorides resulted in a third ring closure. This third ring closure was achieved when an intramolecular nucleophilic attack to the end methylene chloride group was possible. Formation of highly stable five or six membered ring may be counted as a good reason for this ring closure.

Deoxyvasicinone which was previously synthesized by multi step complex reactions (11-15) with long reaction times, harsh reaction conditions or application of microwave irradiation, was easily produced here by application of bi-functional group starting material, «chloroacyl chloride» instead of usual acyl chlorides.

Obtained results of log *p *indicated that the presence of chlorine atom at the end of side chain increased lipophilicity of compounds. In fused pyrazol or pyridazine-quinazolinones, incorporation of NH in hetrocyclic ring reduced liphophilicity of molecules (Table1).

Obtained results of tested compounds against Gram positive bacteria showed that compounds 4, 5 and 10 had the highest activities against *B. subtitles *at 32 or 64 μg/mL concentrations. Results against Gram negative bacteria showed that some compounds showed acceptable activity against *P. aeruginosa*. Existence of a phenyl group at position 3 of quinazolinone and chlorine atom at the end of side chaine in compound 11 could improve activity against *P. aeruginosa *in comparison with compound 9. Anthranilic acid derivatives 2 and 7 with a free carboxylic acid and chlorine atom at the end of side chain showed good activity against *P. aeruginosa *at 32 μg/mL concentration*. *Compounds 3 with benzoxazinone structure and chlorine atom at the end of the side chain also exhibited activity against *P. aeruginosa *at 32 μg/mL concentration. This could be due to the unstability of benzoxazinone ring and possible cleavage to free carboxylic acid derivative. Overall results of antibacterial tests revealed that compounds exhibited better bacteriostatic activity against Gram-negative bacteria. The obtained results of MBC revealed that tested compounds have more significant bacteriostatic than bactericidal activities. Almost all of the screened compounds showed good activity against *C. albicans *and *A. niger*. While Compounds 4 and 9 showed better activities against both fungi at 32 and 64 μg/mL respectively. The obtained results of MFC revealed that tested compounds have more significant fungistatic than fungicidal activities (Tables 2 and 3).

**Table 2:** MIC and MBC results of synthesized compounds against bacteria

**Gram-Negative bacteria MIC, MBC μg/mL**							**Gram-Positive bacteria MIC, MBC μg/mL**						
***S. enteritidis***		***P. aer uginosa***		***E. coli***		***L. monocyto genes***			***B. subtilis***		***S. aureus***		**No.**
**MBC**	**MIC**	**MBC**	**MIC**	**MBC**	**MIC**	**MBC**		**MIC**	**MBC**	**MIC**	**MBC**	**MIC**	
NA	G	256	32	G	512	NA		G	NA	G	NA	G	2
G	512	256	32	G	256	NA		G	NA	G	NA	G	3
G	128	G	128	256	128	NA		G	G	32	G	512	4
NA	G	NA	G	NA	G	NA		G	G	32	NA	G	5
G	512	G	32	G	512	NA		G	NA	G	NA	G	6
NA	G	G	32	G	512	NA		G	G	512	NA	G	7
NA	G	NA	G	NA	G	NA		G	G	512	NA	G	9
NA	G	NA	G	G	256	NA		G	G	64	NA	G	10
NA	G	256	32	G	64	NA		G	G	512	NA	G	11
G	512	NA	G	G	512	NA		G	NA	G	NA	G	12
NA	G	NA	G	G	512	NA		G	NA	G	NA	G	13
**Ciprofloxacine (standard antibacterial agent)**													

G: Growth; MIC: Minimum inhibitory concentration; MBC: Minimum bactericidal concentration; NA: Not applicable

**Table 3:** MIC (μg/mL) and MFC **(**μg/mL**) **results of synthesized compounds against fungi

***A. flavus***		***A. niger***		***C. albicans***		**No.**
**MFC**	**MIC**	**MFC**	**MIC**	**MFC**	**MIC**	
G	256	G	256	512	128	**2**
NA	G	G	512	NA	G	**3**
G	512	G	32	512	32	**4**
G	256	G	128	G	64	**5**
NA	G	G	256	NA	G	**6**
G	256	NA	G	G	512	**7**
G	512	G	64	G	64	**9**
NA	G	G	128	512	32	**10**
NA	G	G	64	G	256	**11**
G	256	G	512	G	128	**12**
G	256	G	128	G	128	**13**
**Ketoconazol ((standard anti fungal agent)**						

G: Growth; MIC: Minimum inhibitory concentration; MFC: Minimum fungicidal concentration; NA: Not applicable
